# The role of compatibility in long-term action-effect binding and effect memory

**DOI:** 10.3758/s13421-025-01741-w

**Published:** 2025-06-20

**Authors:** Marcel R. Schreiner, Viola Mocke, Wilfried Kunde

**Affiliations:** Department of Psychology, Julius-Maximilians-Universität Würzburg, Röntgenring 11, 97070 Würzburg, Germany

**Keywords:** Feature binding, Learning, Memory, Action-effect compatibility, Event file

## Abstract

Through interactions with our environment, we cause perceivable effects. In four experiments, we investigated long-term bindings between action and effect features in action-effect episodes, and how they are influenced by action-effect compatibility (AEC). In addition, we asked whether AEC facilitates memory for effects. In a prime phase, participants performed actions that resulted in an effect that comprised two features, namely a spatial feature (a linear movement or rotation of a box in a certain direction) and a certain identity of a word presented in that box. The effect movement or rotation was either spatially compatible or incompatible (or neutral, in Experiment 3) to the action. In a subsequent probe phase, we assessed whether participants were inclined to repeat the action when re-presented with the effect word. Memory for effect words was also tested. Results showed a higher propensity to repeat an action in Experiments 1–3, but not when the influence of spatial effect features was reduced (Experiment 4). Participants further tended to retrieve the spatial effect location in the neutral condition to a similar extent to in the compatible and incompatible condition in Experiment 3. These findings suggest long-term feature bindings after one-shot learning of action-effect episodes, although primarily due to bindings between different effect features rather than between action and effect features. AEC facilitated memory for effect words in a free recall test (Experiment 1) given repeated presentation of the effect word, but not in a recognition test (Experiments 2–4). Theoretical implications of these findings are discussed.

## Introduction

When we interact with our environment, our actions cause perceivable effects. Various features of such effects are closely linked to the motor activities that produce them. For example, moving an arm leftwards produces a visual trajectory of the arm moving leftwards rather than rightwards. Firmly knocking on a table produces a loud noise rather than a soft one. Blowing into a flute for a long time produces a long tone rather than a short one. Such action-effect linkages have been experienced often enough that the corresponding effects are considered *compatible*. Given this learning history it is no wonder that agents expect to produce compatible effects by their actions, and experience a reduced sense of agency (Haggard, 2017) when incompatible effects occur which violate these expectations (Farrer et al., 2008; Schwarz et al., 2022; Wegner & Wheatley, 1999).

The reoccurrence of a perceptual effect at a later point in time can retrieve the action that produced or co-occurred with the same effect earlier (Dutzi & Hommel, 2009; Elsner & Hommel, 2001; Moeller et al., 2016, 2019). For example, Dutzi and Hommel (2009) used a sequential prime-probe paradigm (i.e., a paradigm in which each trial consists of a prime and a probe episode). Participants first freely chose to press one out of two keys (the action) in a prime, which caused either a low- or a high-pitched tone (the effect). In an immediately following probe, after hearing another tone, participants could again freely choose a key to press. When the tone of the prime repeated in the probe, participants also tended to repeat the prime action more often than when the tone changed. This is presumably due to binding of features associated with the action (such as the location of the action or effector used), and effect produced (such as the pitch of the produced tone, Frings et al., 2020, 2024; Hommel et al., 2001).

According to the theory of event coding (Hommel et al., 2001), environmental events, be they produced by one’s actions or perceived, are represented by feature codes. Feature codes referring to the same event are bound together into a structure called an “event file” (Hommel, 1998, 2004). An event file is therefore a compound of feature codes, each describing an aspect of the event, such as a stimulus involved in the event, a response made to the stimulus, or perceptual effects produced by the response (Frings et al., 2020). Therefore, when a key press has been bound to an effect tone in a prime event-file, repeating this tone in the probe presumably retrieves the bound action.

Until now, such bindings between action and effect features have been mostly investigated for short time scales, typically a few seconds (Dutzi & Hommel, 2009; Hommel, 2004; Moeller & Frings, 2022; Moeller et al., 2016), and feature bindings in general are assumed to be subject to rapid decay (Frings, 2011; Frings et al., 2022; Pastötter et al., 2021). Recently, however, it has been proposed that feature bindings may be related to the formation of associations in long-term memory (Frings et al., 2023). Indeed, action-effect bindings may persist even after longer time periods of several minutes. In studies by Elsner and Hommel (2001) and Hommel et al. (2003), using a blocked prime-probe paradigm (i.e., a paradigm in which a block with all primes is followed by a block with all probes), participants first underwent an acquisition (or prime) phase, in which they learned an action-effect mapping (a left or right key press producing a high- or low-pitched tone) across several action-effect pairings (without receiving specific instructions to attend to the action-effect mapping). In a later test (or probe) phase, the mapping either matched the one learned in the initial acquisition phase or was reversed. Participants exhibited faster responses and chose responses more frequently, if the mapping matched the one learned in the acquisition phase. Note, however, that in these studies, each pairing was presented several times in the acquisition phase.

Schreiner and Kunde (2024) found evidence for action-effect binding after longer time periods of several minutes even after one-time coupling of action and effect, suggesting that action-effect bindings can persist in long-term memory. In one of the experiments, participants pressed a left or right key to produce an effect image in an initial learning phase (without being instructed to memorize the key-image pairings). When participants recognized old effect images in a later test phase and were directly asked about the action they had initially used to produce an effect, participants could indicate the correct action above chance even after several minutes. Notably, the retrieval of the action was only probed after participants identified an effect as old. This procedure may thus primarily probe bindings that exist in declarative memory. However, such bindings may (also) exist in nondeclarative memory.

In the current research, we chose a more indirect way to probe the durability of action-effect bindings after single encounters. Specifically, instead of directly asking participants about the former action-effect mapping, we tested action retrieval by effect repetition by letting participants freely choose one action alternative upon effect re-presentation in the probe. Further, we probed effects irrespective of whether they were correctly recognized or recalled. Moreover, we studied a possible constraint of forming persistent action-effect bindings and retrieving them. For various reasons, the formation or retrieval of such bindings may depend on the compatibility between an action and its associated environmental effect (i.e., action-effect compatibility, AEC): First, incompatible effects may receive less attention leading to reduced binding with the prompting action. Second, effects incompatible with the action contradict established associations between motor patterns and their (typically) compatible effects, thus making it harder to form bindings that run counter to already established associations. Third, even if bindings are established, the system might not retrieve the corresponding response upon perception of an incompatible effect stimulus, or perhaps even prevent such conflict-laden events. Fourth, for incompatible episodes, action and effect features contradict on a shared feature dimension, but not for compatible episodes. To elaborate, not only actions and effect features may be bound, but also different features of the effect. For example, when producing a tone, effect features may entail the frequency, volume, and timbre. When moving an object, effect features may entail the movement direction of the object and its final location.^1^ For action-effect incompatible situations only, this creates the possibility of interference between action-effect bindings and effect-effect bindings.

Previous research already provides initial evidence for such influences of AEC. For instance, AEC has been found to affect declarative memory – specifically, recognition memory for action effects (Hon & Teo, 2025; Hon & Yeo, 2021; Schreiner et al., 2024a; but see Tsuji & Imaizumi, 2022). In a study by Hon and Yeo (2021) participants could move a box by pressing an up or down arrow key. The box then moved either in a direction compatible with the participant’s key press (e.g., upwards if the participant pressed the up arrow key) or incompatible with the participant’s key press (e.g., downwards if the participants pressed the up arrow key). After the box movement, a word appeared superimposed on the box and participants later performed an old/new recognition test with these words and novel foils. Although participants had not been instructed to memorize these words, recognition memory (hit rates) was better for words in compatible action-effect episodes than for words in incompatible ones.

In a sequential prime-probe paradigm, Mocke et al. (in press) investigated the influence of AEC on short-term action-effect bindings. They also let participants perform key presses followed by compatible or incompatible box movements. At the end of the box movement, a word appeared superimposed on the box (the effect word). Each such prime trial was immediately followed by a probe trial. In these probes, effect words were presented superimposed on a box in the screen center and participants could freely decide to press a key. Repeating the effect words retrieved the prime actions (manifested in an increased tendency to repeat the prime action if the effect word was repeated), but more so if the prime episodes were action-effect compatible than incompatible. Thus, at least after a very short delay, re-encountering an effect spontaneously retrieves a prompting action more when action and effect had been compatible.

In addition, the experiments by Schreiner and Kunde (2024) yielded some indication that even after longer delays of several minutes, action-effect bindings were affected by AEC. After participants had experienced each key-press-image pairing once, they were explicitly asked to indicate the key that had initially produced each recognized image. Participants tended to be better able to correctly indicate these keys for images that had appeared in a spatial location compatible (vs. incompatible) to the key.

**Fig. 1:**
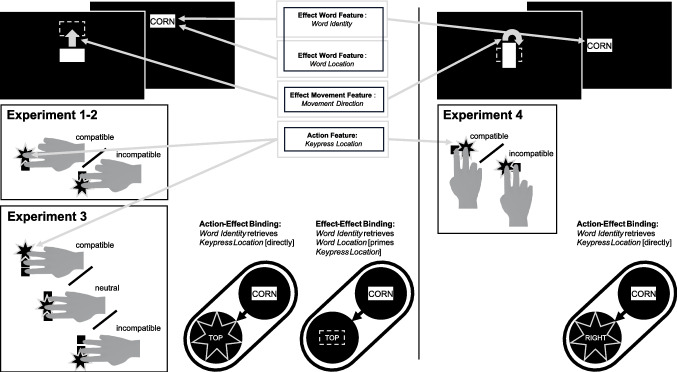
Relevant feature types and possible event files for each experiment. Word location in Experiments 1–3 is equivalent to movement direction (up vs. down). In Experiment 4, movement direction (clockwise vs. counterclockwise) is not linked to the (here: constant) word location. The depicted effect-effect binding serves as a mechanistic explanation for the obtained effect of action-effect compatibility (AEC) in Experiments 1–3. With constant word locations, this binding is not possible in Experiment 4, which explains the lack of an AEC effect

Here, we investigate the effect of AEC on long-term action-effect binding and declarative memory. Therefore, this research directly informs current feature-based action control theories (e.g., Frings et al., 2020) that are based on the Theory of Event Coding (Hommel et al., 2001), and focus on identifying moderators of event file formation, maintenance, and retrieval. Table 1 shows some key characteristics of previous studies, as well as the same characteristics for the current research. The current paradigm works as follows. Through an action (a key press), participants could move a box on the screen (the effect movement). After the box stopped moving, a word (the effect identity) appeared superimposed on the box. The word therefore appeared on a specific location on the screen (the effect location). We therefore distinguish between one action feature (the location of the pressed key) and three effect features (the effect identity, movement, and location). For example, when participants press the up key (action location), the box may move upwards (effect movement), a word appears on the box (effect identity), and is shown in the upper location (effect location). An overview of these different features, as implemented in the current research, is shown in Fig. 1.

**Table 1:** Key characteristics of selected previous studies and of the current research

Study	Type of prime-probe paradigm	Time between prime and probe trials	Explicit instruction to retrieve action in probe?	Number of actions	Number of effects	Repetitions of action-effect episodes	Effect format
Dutzi and Hommel (2009)	Sequential	Few seconds	No	2	2	Not applicable	Tones, colored squares
Elsner and Hommel (2001)	Blocked	Several minutes	No	2	2	≈ 100	Tones
Hommel et al. (2003)	Blocked	Several minutes	No	2	2	≈ 100	Words
Schreiner and Kunde (2024)	Blocked	Several minutes	Yes	2	120	1	Object images
Mocke et al. (in press)	Sequential	Few seconds	No	2	80	1	Words
current	Blocked	Several minutes	No	2–3	80	1	Words

More precisely, we investigate whether AEC facilitates spontaneous response retrieval upon reoccurrence of effect identity, focusing on bindings durable over longer time periods of several minutes. In addition, we investigate whether AEC generally facilitates long-term (declarative) memory for effect stimuli. Importantly, we focus on very early learning stages, that is, after single encounters of action-effect episodes. It can be expected that effects tend to be smaller than when focusing on short-term influences (Mocke et al., in press) or repeated encounters of action-effect episodes (cf. Frings et al., 2023), for example due to decay of bindings or interference. This would also be in line with previous findings (Hon & Yeo, 2021; Schreiner & Kunde, 2024; Schreiner et al., 2024a; Tsuji & Imaizumi, 2022).

To investigate the research questions, we conducted four experiments: In Experiment 1, we tested whether participants’ predisposition to reproduce the action that produced a stimulus in a prime phase in a later probe phase is affected by AEC, using a blocked prime-probe paradigm, while also investigating effects of AEC on free recall performance. Experiment 2 served as a replication and extension of Experiment 1, investigating the effect of AEC on recognition instead of free recall performance and additionally investigating the interplay between response retrieval and recognition. To foreshadow the results, there was overall fairly limited evidence for binding of actions to effect features, while binding among several effect features appeared to occur. In Experiments 3 and 4 we thus aimed to delineate the contribution of different binding types (action-effect or effect-effect) to action selection.

## Experiment 1

In Experiment 1, we employed a blocked prime-probe paradigm. In a prime phase, participants freely decided between two response options (pressing the up or down arrow key on a keyboard) as an action. Each action was followed by a spatially compatible or incompatible effect movement (an upward or downward box movement to the top or bottom of the screen) and a subsequent onset of an effect word (the effect identity) within the box. In a subsequent probe phase, some of these as well as novel words were presented, and participants freely choose one of the actions. Thus, for effect identities that appeared in the prime phase, the responses in the probe phase could either constitute an action repetition (i.e., choosing the same action as in the prime phase) or an action alternation. We hypothesized that compatibility between an action and its effect facilitates action-effect (A-E) binding compared to incompatibility between an action and its effect. We therefore expected that, in the probe phase, there would be an increased proportion of action repetitions for words from compatible trials compared to words from incompatible trials upon presentation of an effect identity from the prime phase (Hypothesis 1).

In a third phase, participants were asked to freely recall words from the prime phase. Here, we hypothesized that (declarative) memory would be better for effect identities that were compatible with the action in the prime phase than for effect identities that were incompatible with the action. We therefore expected free recall performance to be better for words that appeared in compatible trials compared to words that appeared in incompatible trials (Hypothesis 2).

### Method

#### Participants

Participants were recruited via Prolific (https://www.prolific.com) and received a compensation of £2.30 for the approximately 15-min experiment. They were prescreened to be native English speakers and to have normal or corrected-to-normal vision. An a priori power simulation using the R packages *simr* (version 1.0.7, Green & MacLeod, 2016) and *SimDesign* (version 2.1.3, Chalmers & Adkins, 2020) based on data from a previous experiment (Mocke et al., in press) and a pilot study (*N* = 13) yielded a desired sample size of 180 participants to test Hypothesis 1 with a power of 1 − β ≥ 0.85 (for detecting an effect of an unstandardized model coefficient in a generalized mixed linear model analysis of *b* = 0.07) and Hypothesis 2 with a power of 1 − β > 0.99 (for detecting an effect of *b* = 0.15) given a significance level of α = 0.05 (one-tailed testing). Due to possible data exclusion, we oversampled by 15%, thus collecting data from 207 participants. The data of four participants were not transmitted due to technical problems. One participant was excluded due to showing biased action selection in the prime phase (proportion of up arrow key presses larger than 0.80 or lower than 0.20). Another three participants were excluded because effect identities could not be balanced across the cells of compatibility condition × prime action identity in the probe phase. Another ten participants were excluded because they processed less than two arithmetic tasks in a filler phase or failed to respond correctly to any arithmetic task in the filler phase. Another seven participants were excluded because they indicated their data should not be used (e.g., due to distractions). Finally, another participant was excluded due to having experienced technical problems. Thus, the final sample consisted of *N* = 181 participants (*M*_age_ = 38.0 years, *SD*_age_ = 12.4 year; 87 female, two non-binary, nine not disclosing their gender; 153 right-handed, two ambidextrous, nine not disclosing their handedness).

#### Design

The experiment employed a one-factorial (compatibility condition: compatible vs. incompatible) within-subjects design. Dependent variables were action repetition from prime to probe (1 = action repetition, 0 = action alternation) and correct recall of the effect identities (1 = recall, 0 = no recall).

#### Stimuli

Words were 120 English nouns taken from the Glasgow Norms (Scott et al., 2019). The selected nouns were of equal length (four letters), neutral emotional valence (mean ratings between 4.5 and 5.5 on a 9-point scale), and had mean arousal ratings between 3 and 5, mean imageability ratings between 5 and 7, and mean concreteness ratings between 5 and 7 (on a 9-point scale). From the remaining 138 words, we excluded six plural forms and then chose the 120 words that were least ambiguous regarding their word class and most common (see Appendix).

#### Procedure

**Fig. 2:**
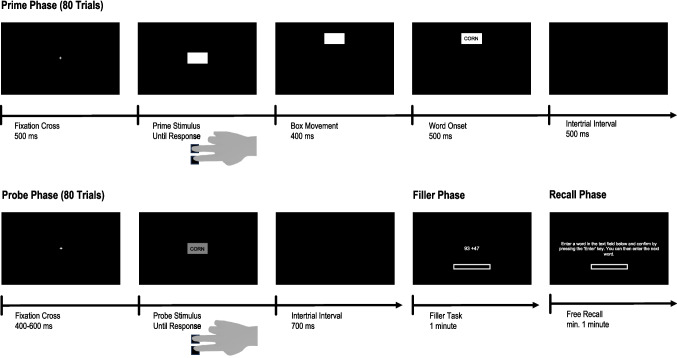
Procedure for Experiment 1

The experiment employed a blocked prime-probe paradigm consisting of a prime phase, which closely followed the procedure used by (Hon & Yeo, 2021, Experiment 2), a probe phase, a filler phase, and a recall phase. The experimental procedure is shown in Fig. 2. The experiment was programmed using PsychoPy (Peirce et al., 2019) and hosted on Pavlovia (https://pavlovia.org/). At the beginning of the experiment, participants gave informed consent and provided basic demographic information.

In the prime phase, participants were presented with a white box on a black background and made self-decided and -initiated up and down arrow key presses (the action). They were instructed to avoid bias in the selection of keys. The box then either moved in the direction indicated by the key press (compatible condition) or in the opposite direction (incompatible condition) for 400 ms (the effect movement). The order of compatible and incompatible trials was randomized. After the box stopped moving at 25% or 75% screen height, a word appeared superimposed on the box for 500 ms (the effect identity). Participants were instructed to remember these words and informed that their memory would later be tested. Each trial began with a 500-ms fixation cross and ended with an intertrial interval of 500 ms during which a blank screen was shown. The prime phase consisted of 80 trials. Eighty words (40 per compatibility condition) were randomly drawn from the pool of 120 words. Thus, there was an equal number of compatible and incompatible trials that occurred in random order. This resulted in an overall unpredictable environment. However, within a given trial, the effect direction could either match or mismatch the participant’s action. Therefore, participants had transient control on the trial-level as opposed to sustained control. While this may lead to weaker overall expectations regarding the effect direction, it allows us to manipulate this expectation on a trial-by-trial basis. In addition, also with such random ordering, participants experience more control in compatible than in incompatible trials (Schreiner et al., 2024a), suggesting this approach to be feasible. Before the prime phase, participants conducted four practice trials (two per compatibility condition) to familiarize them with the task. In the practice trials, the word “word” was used as the stimulus. After the practice trials, participants received feedback on the proportion of their key presses to sensitize them to the instruction of avoiding bias in their key selection.

After the prime phase, the probe phase started. In this phase, participants were presented with words in the screen center superimposed on a gray box and were again instructed to make self-decided and -initiated up and down arrow key presses. They were informed that they no longer had to avoid bias in their key selection. This was done to reduce the likelihood of participants exhibiting specific response strategies, for example alternating their response from the response in a previous trial. First, a fixation cross was presented for a duration randomly jittered between 400 and 600 ms (increments of 10 ms). This was done to prevent participants from rhythmically pressing keys to advance quickly through the trials. Then, a word appeared in the screen center superimposed on a gray box. Upon an action by the participant, the box with the word disappeared. After an intertrial interval of 700 ms the next trial started. The probe phase consisted of 80 trials. Forty words were randomly drawn from the ones presented in the prime phase, with the constraint that the algorithm attempted to balance the number of words across the cells of compatibility condition × prime action identity (i.e., randomly draw ten words each from words in the compatible condition in trials with an “up” action, words in the compatible condition in trials with a “down” action, words in the incompatible condition in trials with an “up” action, and trials in the incompatible condition in trials with a “down” action). If this was not possible, words were only balanced across the two compatibility conditions (i.e., 20 words were randomly drawn from the congruent and incongruent condition), and the respective participants were flagged and excluded from the analyses. The other 40 words were new words that did not appear in the prime phase. Responses to these new words were used to derive a person-specific baseline to account for possible biases in response selection (see *Data analysis* section). Trial order was randomized.

After the probe phase, participants conducted a filler task during which they had to solve randomly generated arithmetic tasks for 1 min to avoid recency effects. After the filler task, participants conducted an intentional free recall test. They were asked to recall as many words from the prime phase as possible and had to spend at least 1 min trying to recall words. At the end of the experiment, participants could indicate whether there are any reasons why their data should be excluded from the analyses and provide further comments regarding the experiment.

#### Data analysis

##### Action repetitions

For testing Hypothesis 1, we determined whether the key press given as a response to a specific word in the probe phase was the same key press that produced that word in the prime phase (action repetition) or a different key press (action alternation). Further, to account for the influence of a biased response selection in the probe phase, we calculated a bias index (BI) reflecting the proportion of the more frequently chosen key to the less frequently chosen key for responses to new words (i.e., words that did not occur in the prime phase). This index shows the deviation from an equal proportion (0.5) of key selection and can thus vary between 0 and 0.5. It is calculated as:

1 $$\mathrm{BI}=\left|0.5-\frac{{n}_{up}}{{n}_{tot}}\right|,$$

where *n*_*up*_ is the number of up key responses and *n*_*tot*_ is the total number of responses in the probe phase.

The hypothesis was then tested by fitting a generalized mixed linear model (see Goldstein, 2011) with a logit link function, using action repetition (1 = action repetition, 0 = action alternation) as the binary dependent variable. The analysis was thus conducted on the trial instead of on the aggregate level (see Hoffman & Rovine, 2007). Mixed linear models provide a number of advantages to more traditional analyses based on aggregated data, such as *t*-tests and analyses of variance, for example by being able to yield higher statistical power and to better account for dependencies present in the data (e.g., Aarts et al., 2014; Hoffman & Rovine, 2007). In addition, running analyses based on aggregates of response repetitions may be problematic, an issue that we discuss in more detail in the *Results* section.^2^ Compatibility condition served as the independent variable (effect-coded, 1 = compatible, −1 = incompatible) and the BI as a control variable (fixed effects). The model further included random person intercepts to account for repeated measurement.^3^ Note that a model including random slopes for compatibility condition yielded a slightly worse fit as indicated by the difference in the Bayesian Information Criteria (ΔBIC; negative values indicate better fit of the model without random slopes) of the two models (ΔBIC = −17).

##### **Recall performance**

For testing Hypothesis 2, we determined for each effect identity in the prime phase whether the word was successfully recalled in the free recall test. The hypothesis was then also tested by fitting a generalized mixed linear model with a logit link function. The model further included random person intercepts and random word intercepts. A model with random slopes for compatibility condition again yielded slightly worse fit (ΔBIC = −22.9). Correct recall (1 = recall, 0 = no recall) served as the binary dependent variable and compatibility condition (effect-coded) served as the independent variable. Because some words could occur both in the prime and the probe phase, we controlled for these differences in exposure by including the factor word repetition (effect-coded, 1 = repetition, −1 = no repetition), as well as its interaction with compatibility condition, as a control variable in the model.

As our hypotheses were directed, the critical tests for an effect of compatibility condition were one-tailed and we report one-sided *p* values (*p*_*os*_). Tests for the control variables were two-tailed and we report two-sided *p* values (*p*_*ts*_). We further report two-sided 95% Wald confidence intervals (CIs) for fixed effects. Models were fit using the R packages *lme4* (version 1.1–35.1, Bates et al., 2015) and *lmerTest* (version 3.1–3, Kuznetsova et al., 2017).

##### Exploratory analysis: Recall performance and action repetitions

We further conducted an exploratory analysis to examine whether the tendency to repeat the action that produced a certain effect identity upon presentation of that word in the probe phase and later memory retrieval of the effect identity are not independent, but positively correlated. Thus, we investigated whether free recall performance was better for words that were followed by an action repetition in the probe phase than for words that were followed by an action alternation and whether this effect differed between the compatible and incompatible condition. For this analysis we only considered words that appeared in both the prime and the probe phase. Here we fit a generalized mixed linear model with a logit link function and random person and word intercepts. A model with random slopes of compatibility condition again yielded slightly worse fit (ΔBIC = −17.3). Correct recall was the dependent variable and compatibility condition (effect-coded) and action repetition (effect-coded, 1 = action repetition, −1 = action alternation), as well as their interaction, were the independent variables.

### Results

#### Action repetitions

**Fig. 3:**
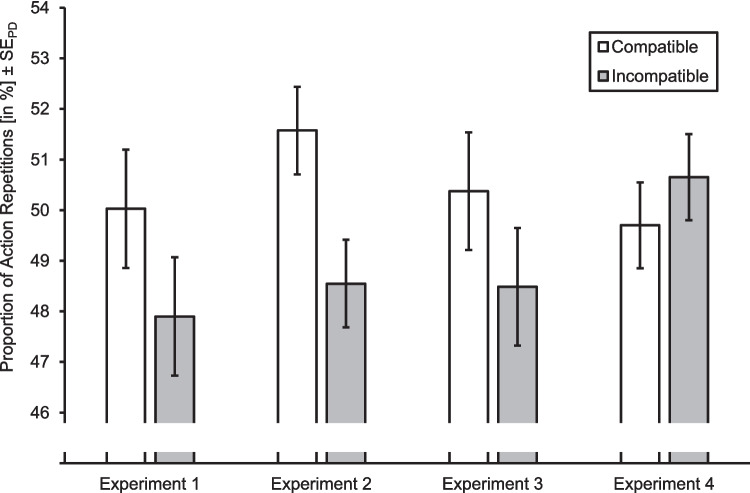
Proportion of action repetitions by compatibility condition in all experiments. Error bars represent ± standard error of paired differences (see Pfister & Janczyk, 2013). For Experiment 3, the neutral compatibility condition is not displayed

For testing Hypothesis 1, we tested the effect of compatibility condition on action repetitions in the probe phase. The proportion of action repetitions by compatibility condition is depicted in Fig. 3. Note that, while we report these statistics for completeness and illustration purposes, the group-level means do not necessarily give an accurate indication of action-effect binding. This is because the individual baseline (or chance level) is not necessarily 0.5 due to participants being able to freely select their actions in both the prime and the probe phase. Specifically, if a participant favors a certain response key to a different extent in the prime than in the probe phase, the individual baseline will differ from 0.5.^4^ If, for example, a participant favors one of the keys in the prime trials, but the other key in the probe trials, this would naturally create an action repetition level smaller than 0.5 (e.g., 0.45). If the participant exhibited a proportion of action repetitions in the probe phase of 0.48, this would be indicative of action-effect binding but would draw the group-level mean towards a value smaller than 0.5. A trial-level model-based analysis therefore yields better interpretable results. The generalized mixed linear model analysis revealed a significant effect of compatibility condition (*b* = 0.04, 95% CI [−0.00, 0.09], *z* = 1.81, *p*_*os*_ = 0.035). The proportion of action repetitions was higher in the compatible (*M* = 0.50, *SD* = 0.11) than in the incompatible (*M* = 0.48, *SD* = 0.11) condition. Therefore, Hypothesis 1 was supported. There was no effect of the BI (*b* = 0.19, 95% CI [−0.13, 0.52], *z* = 1.18, *p*_*ts*_ = 0.24).

#### Recall performance

**Fig. 4:**
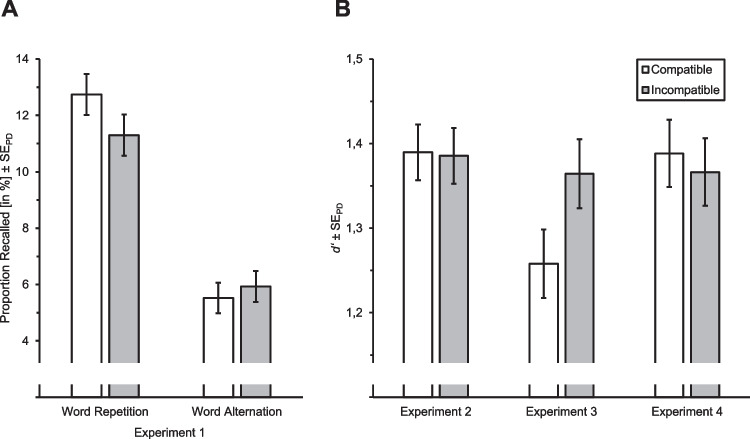
Memory performance. (**A**). Proportion of recalled words by compatibility condition and word repetition in the free recall test in Experiment 1. (**B**) Mean *d’* values by compatibility condition in the recognition test in Experiments 2–4. *Notes*. Error bars represent ± standard error of paired differences (see Pfister & Janczyk, 2013). For Experiment 1 (**A**), standard errors were computed separately for the word repetition conditions. For Experiment 3, the neutral compatibility condition is not displayed (**B**)

For testing Hypothesis 2, we tested the effect of compatibility condition on free recall performance. The proportion of action repetitions by compatibility condition and word repetition is depicted in Fig. 4A. The generalized mixed linear model analysis revealed no significant main effect of compatibility condition (*b* = 0.02, 95% CI [−0.04, 0.08], *z* = 0.60, *p*_*os*_ = 0.28). The proportion of recalled words did not differ between the compatible (*M* = 0.09, *SD* = 0.07) and incompatible (*M* = 0.09, *SD* = 0.06) condition. However, there was a significant main effect of word repetition (*b* = 0.42, 95% CI [0.36, 0.48], *z* = 13.54, *p*_*ts*_ < 0.001), with a higher proportion of recalled words if they were repeated in the probe phase (*M* = 0.12, *SD* = 0.10) than if they were not repeated and only shown in the prime phase (*M* = 0.06, *SD* = 0.05). Notably, there was also a significant interaction between word repetition and compatibility condition (*b* = 0.06, 95% CI [0.00, 0.12], z = 2.03, *p*_*ts*_ = 0.043). Post-hoc pairwise comparisons using the R package *emmeans* (version 1.8.9, Lenth, 2023) revealed that the difference in recall performance between the compatible and incompatible condition was significant for words that were repeated in the probe phase (log odds ratio [log OR] = 0.16, 95% CI [0.02, 0.31], *z* = 2.22, *p*_*ts*_ = 0.026). The proportion of recalled words was higher in the compatible (*M* = 0.13, *SD* = 0.11) than in the incompatible (*M* = 0.11, *SD* = 0.10) condition if words were repeated. However, if words only occurred in the prime phase and were thus not repeated, recall performance did not significantly differ between the compatible (*M* = 0.06, *SD* = 0.06) and the incompatible (*M* = 0.06, *SD* = 0.06) condition (log OR = −0.09, 95% CI [−0.29, 0.11], *z* = −0.89, *p*_*ts*_ = 0.38). Apparently, AEC did only affect recall of words that were sufficiently well encoded due to being presented twice (once in the prime and once in the probe phase). Thus, Hypothesis 2 was only partially supported.

#### Exploratory analysis: Recall performance and action repetitions

For our exploratory analysis, we tested the effect of compatibility condition and action repetition on free recall performance. The generalized mixed linear model analysis revealed a significant main effect of compatibility condition (*b* = 0.08, 95% CI [0.01, 0.15], *z* = 2.20, *p*_*ts*_ = 0.028). This is consistent with the finding that recall performance was better in the compatible than in the incompatible condition for words that were presented twice. There was neither a significant main effect of action repetition (*b* = 0.02, 95% CI [−0.05, 0.09], *z* = 0.58, *p*_*ts*_ = 0.60), nor a significant interaction (*b* = −0.03, 95% CI [−0.11, 0.04], *z* = −0.93, *p*_*ts*_ = 0.35).

### Discussion

In Experiment 1, we found evidence for a higher propensity for participants to repeat an action from a previous prime phase in a later probe phase if they are re-presented with an effect identity that followed an effect movement which was compatible rather than incompatible with the participant’s action in the prime phase. This, at first glance, supports Hypothesis 1 and suggests that AEC (i.e., the compatibility between actions and effect movements) facilitates binding of actions and effect identity. Notably, as we employed a blocked prime-probe paradigm, these one-shot bindings still existed after longer durations, thus appearing to be quite durable.

Besides this memory for features of an action-effect episode, we also found some evidence for AEC to affect long-term declarative memory. Specifically, we found better free recall performance for effect identities occurring at positions that were compatible with the participants’ actions than for effect identities occurring at positions incompatible with the participants’ actions in the prime phase. However, this was only the case if these effect identities additionally appeared in the probe phase and were thus presented twice to the participants. This suggests that effect identities need to be sufficiently well encoded for a compatibility effect to emerge and that encoding is facilitated by the repeated presentation of the effect identities. For effect identities only presented in the prime phase, recall performance was much poorer and the effect may therefore have been masked due to floor effects. The obtained effect of AEC on effect identity recall is consistent with Hon and Yeo (2021) and Schreiner et al. (2024a), who found a facilitating effect of AEC on long-term memory using a recognition memory test. However, in an exploratory analysis we found no effect of action retrieval in the probe phase on long-term memory. That is, free recall performance was independent of whether participants repeated or alternated their action from the prime phase in the probe phase. Apparently, the strength of the action-word link, which drives the effect of action retrieval by effect identity repetition, and the strength of the memory for the effect identity, which drives the free recall performance, are independent. This may be due to the free recall test providing no cue to retrieving an associated action in the recall phase, and therefore action-effect associations may not help with the retrieval of the effect identity in an unguided memory search.

In Experiment 2, we used a recognition instead of a free recall test, for which the effect identities are also re-presented in the memory test. In this case, action retrieval may be more helpful for memory performance by improving recognition (i.e., the successful retrieval of an action associated with a stimulus may help the identification of that stimulus as having been previously encountered). With this experiment we further set out to replicate the finding of highly durable A-E bindings.

## Experiment 2

Experiment 2 served as a replication and extension of Experiment 1, using an old/new recognition instead of a free recall test to assess the effect of AEC on declarative long-term memory. Furthermore, we investigated the previously only exploratorily probed interplay between action retrieval and recognition in a preregistered analysis. Specifically, we expected better recognition performance for words that were followed by an action repetition in the probe phase than for words that were followed by an action alternation (Hypothesis 3) and tested whether this effect was more pronounced for compatible than for incompatible trials.

### Method

#### Participants

Participants were recruited based on the same criteria as in Experiment 1 and were not to have participated in the previous experiment. An a priori power simulation using the R packages *simr* (version 1.0.7, Green & MacLeod, 2016) and *SimDesign* (version 2.1.3, Chalmers & Adkins, 2020) based on the data from Experiment 1 and the pilot study (*N* = 13) yielded a desired sample size of *N* = 175 participants to test Hypotheses 1 and 3 with a power of 1 − β > 0.90 given a significance level of α = 0.05 (one-tailed testing). An a priori power analysis using the package *pwr* (version 1.3–0, Champely, 2020) further showed that this sample size allows us to test Hypothesis 2 with a power of 1 − β > 0.90 given an effect size of *d* = 0.22 (one-tailed testing). Due to possible data exclusion, we oversampled by 15%, thus collecting data from 202 participants. The data of four participants were not transmitted due to technical problems. Three participants were excluded due to showing biased action selection in the prime phase (proportion of up arrow key presses larger than 0.80 or lower than 0.20). Another six participants were excluded because they processed less than two arithmetic tasks or failed to respond correctly to any task in the filler phase. Finally, another three participants were excluded due to having experienced technical problems. Thus, the final sample consisted of *N* = 186 participants (*M*_age_ = 40.3 years, *SD*_age_ = 12.4 years; 90 female, one non-binary, seven not disclosing their gender; 148 right-handed, two ambidextrous, seven not disclosing their handedness).

#### Design, stimuli, and procedure

Dependent variables were action repetition from prime to probe (1 = action repetition, 0 = action alternation), correct recognition of the effect identities (1 = recognition, 0 = no recognition), and the sensitivity index *d’* from signal detection theory. The independent variables and stimuli were identical to Experiment 1. The procedure was also identical to Experiment 1, with the following exceptions: First, the probe phase contained all 80 words from the prime phase and no new words (considering the non-significant effect of the bias index in Experiment 1). This also increased the power for testing Hypothesis 1, as more relevant trials were available per participant. Second, instead of an intentional free recall test, we employed an intentional old/new recognition test. In this recognition phase, participants were presented with words in the screen center and were asked to determine whether the word is old (i.e., was shown in the prime and probe phase) or new. The recognition phase consisted of 80 trials. Forty old words were randomly drawn from the ones presented in the prime and probe phase, with the constraint that the algorithm attempted to balance the number of words across the cells of compatibility condition × prime action identity. If this was not possible, words were only balanced across the two compatibility conditions, and the respective participants were flagged and excluded from the analyses. The other 40 words were new words that were not shown during the prime and probe phase. Thus, the number of old and new words was counterbalanced in the recognition phase. Each trial terminated upon a valid response, followed by a 250-ms intertrial interval. Trial order was randomized.

#### Data analysis

##### **Action repetitions**

Hypothesis 1 was tested as in Experiment 1, except that there was no bias index computed and included as a covariate in the model. A model with random slopes for compatibility condition yielded slightly worse fit (ΔBIC = −18).

##### **Recognition performance**

For testing Hypothesis 2, we computed the sensitivity index *d’* from signal detection theory for each participant from their hit and false alarm rates, separately for the compatible and incompatible conditions. As there were participants with extreme proportions of hit or false alarm rates (i.e., 0 or 1), for which *d’* cannot be computed, we applied the correction suggested by Hautus (1995), while accounting for the relative proportion of signal and noise trials (see also Schreiner et al., 2024a). Individual *d’* values were then subjected to a paired-samples *t-*test with compatibility condition as the independent variable (one-tailed testing). The effect size was computed using the R package *effectsize* (version 0.8.6, Ben-Shachar et al., 2020).

##### Recognition performance and action repetitions

For testing Hypothesis 3, we followed a similar approach as in the exploratory analysis of recall performance and action repetitions in Experiment 1. Only old words in the recognition phase were considered for this analysis. We fit a generalized mixed linear model with a logit link function and random person and word intercepts. A model with random slopes for compatibility condition again yielded slightly worse fit (ΔBIC = −17.3). Recognition performance (hit = 1, miss = 0) was the dependent variable and compatibility condition and action repetition (both effect-coded), as well as their interaction, were the independent variables.

### Results

#### Action repetitions

For testing Hypothesis 1, we tested the effect of compatibility condition on action repetitions in the probe phase. The proportion of action repetitions by compatibility condition is depicted in Fig. 3. The generalized mixed linear model analysis revealed a significant effect of compatibility condition (*b* = 0.06, 95% CI [0.03, 0.09], *z* = 3.69, *p*_*os*_ < 0.001). The proportion of action repetitions was higher in the compatible (*M* = 0.52, *SD* = 0.08) than in the incompatible (*M* = 0.49, *SD* = 0.08) condition. Therefore, Hypothesis 1 was supported.

#### Recognition performance

For testing Hypothesis 2, we tested the effect of compatibility condition on recognition performance. Mean *d’* values by compatibility condition are depicted in Fig. 4B. Recognition performance was not significantly better in the compatible (*M* = 1.39, *SD* = 0.83) than in the incompatible (*M* = 1.39, *SD* = 0.82) condition (Δ*M* = 0.004, 95% CI [−0.06, 0.07], *t*(185) = 0.13, *p*_*os*_ = 0.45, *d* = 0.01, 95% CI [−0.13, 0.15]). Thus, Hypothesis 2 was not supported.

#### Recognition performance and action repetitions

For testing Hypothesis 3, we tested the effect of compatibility condition and action repetition on recognition performance. The generalized mixed linear model analysis revealed neither a significant main effect of compatibility condition (*b* = −0.01, 95% CI [−0.07, 0.05], *z* = −0.44, *p*_*ts*_ = 0.66), nor a significant main effect of action repetition (*b* = 0.02, 95% CI [−0.04, 0.08], *z* = 0.70, *p*_*ts*_ = 0.248, nor a significant interaction (*b* = −0.01, 95% CI [−0.06, 0.05], *z* = −0.20, *p*_*ts*_ = 0.84). Thus, Hypothesis 3 was not supported.

## Discussion

In Experiment 2, we could replicate the finding that participants have a higher propensity to repeat an action from a previous prime phase in a later probe phase if they are re-presented with an effect identity that occurred at a position compatible rather than incompatible with the participant’s action in the prime phase. This again supports Hypothesis 1 and suggests that AEC (i.e., compatibility between action and effect movement) facilitates binding between action and effect identity. However, we did not find evidence for an effect of AEC on long-term declarative memory for effect identities when using a recognition test, nor an effect of action repetition in the probe phase on long-term memory (see also *General discussion*).

So far, we interpreted the effect of AEC on action repetitions as indicating that AEC facilitates A-E binding (i.e., supporting Hypothesis 1). However, there exists an alternative explanation for this observation: Instead of the effect identity retrieving merely the associated action, it is possible that it (also) retrieves other effect features, such as the movement direction of the box (i.e., the effect movement) or its ending position, which is equivalent to the location of the effect identity (i.e., effect location). In fact, it even seems likely that effect identities and their spatial locations become bound via effect-effect (E-E) bindings (see Fig. 1). Participants may then select their action in the probe phase not (only) based on a retrieved spatial action feature (i.e., the key location), but select it to match the retrieved spatial effect feature (i.e., the effect location). For compatible trials, both the retrieval of the spatial action and effect features prompt an action repetition. For incompatible trials however, the retrieval of the spatial action feature prompts an action repetition while the retrieval of the spatial effect feature prompts an action alternation. Thus, the effect of AEC on action repetitions may alternatively be explained by E(identity)-E(location) bindings. The observed effect of AEC may thus not be an effect proper but instead be attributable to E-E- bindings. Findings by Schreiner and Kunde (2024) suggest that participants retrieve effect features and adjust their response even when being explicitly instructed to retrieve the action used to produce a certain effect (see also Dewey & Carr, 2012). The findings further suggest that the effect of AEC on action repetitions likely reflects a mixture of A-E(identity) and E(identity)-E(location) bindings, with effects of E-E bindings being stronger. In Experiment 3, we aimed to delineate the influence of these two types of bindings, which may be responsible for the influence of AEC on action repetitions.

## Experiment 3

In Experiment 3, we aimed to address the problem that the influence of AEC on action repetitions can be explained by either A-E(identity) or E(identity)-E(location) bindings (see Fig. 1). To this end, we included a neutral action alternative in the prime phase (“middle” key location) in addition to the existing alternatives (“top” and “bottom” key locations) that did not overlap with any spatial effect feature (“top” or “bottom” effect location). This neutral condition allowed us to test if the observed compatibility effects may be explained solely by retrieval of spatial effect features (i.e., effect location) while ruling out retrieval of an action followed by a spatial effect. Pressing the middle key in the prime phase still resulted in an upwards or downwards movement of the box, but these prime episodes did not carry any compatibility information due to the neutral spatial position of the key. In the probes, we allowed only top and bottom key presses. For compatible and incompatible conditions, the rate of responses that may reflect retrieval of the action or effect location are linearly dependent. Specifically, in the compatible condition, both retrieval types result in the same outcome. In the incompatible condition, they result in complementary outcomes (i.e., the rate of responses that might reflect retrieval of the effect direction is 1 – the rate of action repetitions). In contrast, probe responses in the neutral condition cannot reflect retrieval of the prime action and therefore allow us to specifically test for the retrieval of effect features.

If effect identity retrieved the effect location in the prime, and participants just responded in the probe in a manner compatible with that retrieved effect location, then matches between prime effect location and probe responses should occur equally often irrespective of which action had produced that effect in the prime, be it an action that was compatible, neutral, or incompatible with that effect location. If, however, prime actions became directly retrieved by effect identity, then matches between effect location and chosen probe response should occur most often when that effect identity had been produced by a compatible action (because a response is retrieved that did match the prime effect location), lower in neutral trials (because no response can be retrieved), and lowest in incompatible trials (because a response is retrieved that did decidedly mismatched the prime effect location) (Hypothesis 4).

### Method

#### Participants

As in Experiment 2, our target sample size was *N* = 175 participants. We again oversampled by 15% to account for possible data exclusions, thus collecting data from 202 participants. An a priori power simulation using the R packages *simr* (version 1.0.7, Green & MacLeod, 2016) and *SimDesign* (version 2.1.3, Chalmers & Adkins, 2020) based on the data from Experiment 2 and an a priori power analysis using the package *pwr* (version 1.3–0, Champely, 2020) showed that this sample size allows us to test Hypotheses 1 and 4 with a power of 1 − β > 0.90. and to detect an effect size of *d* = 0.22 with a power of 1 − β > 0.90 for testing Hypothesis 2 (one-tailed testing). Power for testing Hypothesis 3 was rather low (about 1 − β = 0.60), but as the test of this hypothesis was not the focus of the experiment and considering resource constraints, we deemed this acceptable. The data for one participant was not transmitted due to technical problems. Ten participants were excluded due to showing biased action selection in the prime phase (proportion of pressing any key larger than 0.63 or lower than 0.03). Another ten participants were excluded because words could not be balanced across compatibility conditions in the recognition phase. Another five participants were excluded because they processed less than two arithmetic tasks or failed to respond correctly to any task in the filler phase. Finally, another five participants were excluded due to having experienced technical problems or experienced distractions. Thus, the final sample consisted of *N* = 171 participants (*M*_age_ = 39.3 years,^5^*SD*_age_ = 13.0 years; 83 female, two non-binary, three not disclosing their gender; 144 right-handed, three ambidextrous, two not disclosing their handedness).

#### Design, stimuli, and procedure

The experiment employed a one-factorial (compatibility condition: compatible vs. incompatible vs. neutral) within-subjects design. The dependent variables were identical to the ones in Experiment 2. Stimuli were identical to those of Experiments 1 and 2, except that we excluded one word. Thus, stimuli consisted of 119 English nouns. This number of stimuli allowed for the same number of old and new words in the recognition test while counterbalancing old words across the three compatibility conditions.

**Fig. 5:**
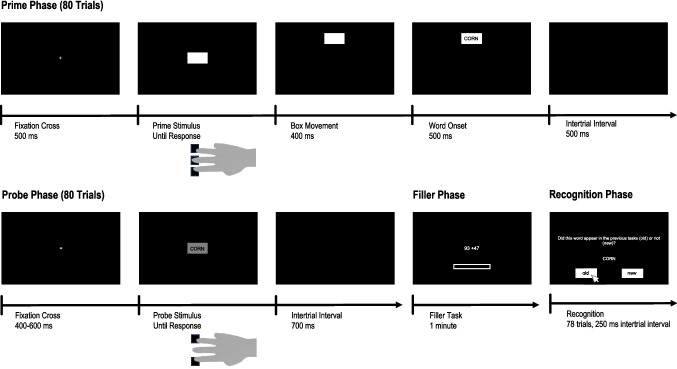
Procedure for Experiment 3. In Experiment 3, three response keys were used in the prime phase. The resulting box movements (up vs. down) had equal probability. In the probe phase, only the top and bottom keys were available. As a test of declarative word memory, Experiments 2–4 employed a recognition test instead of a recall test, as employed in Experiment 1

The procedure was similar to that of Experiment 2 (see Fig. 5). However, in the prime phase, participants could choose between three keys (*U*, *H*, and *B*), which were associated with a top, middle, and bottom action, respectively, based on their location on a QWERTY-based keyboard. Upon a key press, the box moved randomly upwards or downwards (movement condition). For top and bottom key presses, this either led to a movement and later appearance of the word in the direction indicated by the key press (compatible condition), or in the opposite direction (incompatible condition). For middle key presses, the box movement and word location could not overlap with the key location (neutral condition).^6^ The prime phase again consisted of 80 trials. Eighty words (on average, 26.67 trials per compatibility condition) were randomly drawn from the pool of 119 words. Before the experimental trials, participants underwent six practice trials.

In the probe phase, participants could only respond with the top or bottom key. Thus, action repetitions were not possible in the neutral condition. The probe phase again consisted of 80 trials and included the same words as the prime phase, thus including the words from the congruent, incongruent, and neutral condition.

The recognition phase was identical to Experiment 2 but only consisted of 78 trials. Thirty-nine old words were randomly drawn from the ones presented in the prime and probe phase. The algorithm attempted to balance the number of words across the three compatibility conditions (i.e., draw 13 old words per compatibility condition). If this was not possible, words were drawn completely randomly, and these participants were flagged and excluded from the analyses. The other 39 words were new words that were not shown during the prime and probe phase.

#### Data analysis

For testing Hypotheses 1, 2, and 3, the analyses were the same as in Experiment 2. For testing these hypotheses, the data from the neutral condition was excluded. Models with random slopes for compatibility condition yielded slightly worse fit than models with only random intercepts (ΔBIC = −15 for the test of Hypothesis 1 and ΔBIC = −16.3 for the test of Hypothesis 3).

Hypothesis 4 was tested using a generalized mixed linear model with a logit link function. Action match in the probe phase with effect location in the prime phase served as the binary dependent variable (1 = location match, 0 = location mismatch). We conducted planned comparisons by contrast coding the compatibility conditions using two orthogonal contrasts, which served as the independent variables. The first contrast reflects the comparison between the neutral and the other two conditions (with weights 1, 1, −2 for the compatible, incompatible, and neutral conditions, respectively). The second contrast reflects the comparison between the compatible and incompatible conditions (with weights 1, −1, 0). The model further included random person intercepts. A model with random slopes for the planned contrasts yielded worse fit (ΔBIC = −44).

### Results

#### Action repetitions

For Hypothesis 1, we tested the effect of compatibility condition (excluding the neutral condition) on action repetitions in the probe phase. The proportion of action repetitions by compatibility condition is depicted in Fig. 3. The generalized mixed linear model analysis revealed a significant effect of compatibility condition (*b* = 0.05, 95% CI [0.00, 0.09], *z* = 2.12, *p*_*os*_ = 0.017). The proportion of action repetitions was higher in the compatible (*M* = 0.50, *SD* = 0.10) than in the incompatible (*M* = 0.48, *SD* = 0.11) condition. Therefore, Hypothesis 1 was supported.

#### Match of effect location and probe action

For Hypothesis 4, we tested the influence of the compatibility condition on the proportion of probe responses that matched the location (top or bottom) of the prime effect location (i.e., location matches). A location match occurred if a participant selected a response in the probe phase that matched the effect location (or effect movement) of the word in the prime phase, irrespective of whether this response matched the action through which the word was previously produced. The proportion of location matches was *M* = 0.50 (*SD* = *0.10*) in the compatible condition, *M* = 0.52 (*SD* = *0.11*) in the incompatible condition, and *M* = 0.51 (*SD* = *0.09*) in the neutral condition. The generalized mixed linear model analysis revealed that location matches in the neutral condition were not significantly reduced compared to the other two conditions (*b* = −0.00, 95% CI [−0.03, 0.02], *z* = −0.31, *p*_*os*_ = 0.62). In addition, location matches also did not significantly differ between the compatible and incompatible conditions (*b* = −0.02, 95% CI [−0.07, 0.02], *z* = −1.11, *p*_*ts*_ = 0.28). Together, this corroborates the idea of no (relevant) influence of A-E(identity) bindings on the effect of AEC on action repetitions.

#### Recognition performance

For Hypothesis 2, we tested the effect of compatibility condition (excluding the neutral condition) on recognition performance. Mean *d’* values by compatibility condition are depicted in Fig. 4B. Recognition performance was not significantly better in the compatible (*M* = 1.26, *SD* = 0.82) than in the incompatible (*M* = 1.36, *SD* = 0.82) condition (Δ*M* = −0.11, 95% CI [−0.19, −0.03], *t*(170) = −2.62, *p*_*os*_ = 0.995, *d* = −0.20, 95% CI [−0.35, −0.05]). Thus, Hypothesis 2 was not supported. In the neutral condition, *d’* was on average 1.33 (*SD* = 0.88).

#### Recognition performance and action repetitions

For Hypothesis 3, we tested the effect of compatibility condition (excluding the neutral condition) and action repetition on recognition performance. The generalized mixed linear model analysis revealed neither a significant main effect of compatibility condition (*b* = −0.07, 95% CI [−0.15, 0.00], *z* = −1.94, *p*_*ts*_ = 0.053), nor a significant main effect of action repetition (*b* = −0.01, 95% CI [−0.09, 0.06], *z* = −0.38, *p*_*ts*_ = 0.71), nor a significant interaction (*b* = −0.02, 95% CI [−0.10, 0.06], *z* = −0.55, *p*_*ts*_ = 0.58). Thus, Hypothesis 3 was not supported.

### Discussion

In Experiment 3, we yet again found that participants have a higher propensity to repeat an action from a previous prime phase in a later probe phase if they are re-presented with an effect identity that occurred at a location compatible rather than incompatible with the participant’s action in the prime phase. While this may suggest that AEC facilitates A-E(identity) binding, the finding that participants did not exhibit a different tendency to select actions matching the prime effect location depending on the compatibility conditions suggests that the former finding is, at least partly, attributable to E(identity)-E(location) binding (i.e., participants retrieving the effect location and accordingly selecting their action in the probe phase). Note, however, that this conclusion rests on a null effect.^7^ As in Experiment 2, we did not find evidence for an effect of AEC on long-term declarative memory for effect identities, nor an effect of action repetition in the probe phase on long-term memory.

The findings of Experiment 3 suggest that the influence of AEC on action repetitions is at least partly driven by E-E binding besides A-E binding. However, it remains unclear whether E-E binding can fully account for the effect, or whether the effect is driven by a mixture of A-E and E-E binding. In Experiment 4, we followed up on these questions by aiming to reduce the influence of spatial effect features.

## Experiment 4

In Experiment 4, we aimed to disentangle the influence of A-E(identity) and E(identity)-E(location) bindings on the effect of AEC on action repetitions. To this end, we aimed to reduce the influence of spatial effect features by changing the nature of the effect movement in the prime phase. Instead of choosing a vertical box movement, in Experiment 4, participants could choose the rotation direction (clockwise or counterclockwise) of the box, with the effect again being either compatible or incompatible with their key press. At least for very short delays, Mocke et al. (in press) could already demonstrate an effect of compatibility on the retrieval of A-E bindings using the same compatibility manipulation.

With this change, the location of the word on the screen was kept constant across the two compatibility conditions (i.e., in the screen center). In addition, the rotation direction of the box is likely a less salient spatial feature than the movement direction of the box in the previous experiments. As the account based on E-E binding rests on the retrieval of these spatial effect features, the influence of AEC on action repetitions should be substantially reduced or even eliminated in Experiment 4 if this account is valid.

### Method

#### Participants

The sample size was based on the one in Experiment 2. Therefore, our target sample size was again 175 participants and we oversampled by 15% to account for possible data exclusions, thus collecting data from 202 participants. The data of five participants were not transmitted due to technical problems. One participant was excluded due to showing biased action selection in the prime phase (proportion of pressing either of the two response keys larger than 0.80 or lower than 0.20). Another two participants were excluded because words could not be balanced across the cells of compatibility condition × prime action identity in the recognition phase. Another nine participants were excluded because they processed less than two arithmetic tasks or failed to respond correctly to any task in the filler phase. Finally, another nine participants were excluded due to having experienced technical problems or had problems focusing on the study. Thus, the final sample consisted of *N* = 176 participants (*M*_age_ = 40.5 years, *SD*_age_ = 14.0 years; 83 female, two non-binary, six not disclosing their gender; 146 right-handed, two ambidextrous, eight not disclosing their handedness).

#### Design, stimuli, procedure, and data analysis

The design and stimuli were identical to Experiment 2. The procedure was also identical to Experiment 2, with the following exceptions: In the prime phase, participants could indicate whether the box should move clockwise (by pressing the M key) or counterclockwise (by pressing the N key). To facilitate this action-effect mapping, an image depicting the mapping was shown below the initially vertically oriented box. The image disappeared after a key was pressed. The box then either rotated in the direction indicated by the key press (compatible condition) or in the opposite direction (incompatible condition) until it reached a horizontal orientation. The box movement lasted 500 ms. Analyses were the same as in Experiment 2. Models with random slopes for compatibility condition (and response repetitions for Hypothesis 3) yielded slightly worse fit than models with only random intercepts (ΔBIC = −19 for the test of Hypothesis 1 and ΔBIC = −39.8 for the test of Hypothesis 3).

### Results

#### Action repetitions

For Hypothesis 1, we tested the effect of compatibility condition on action repetitions in the probe phase. The proportion of action repetitions by compatibility condition is depicted in Fig. 3. The generalized mixed linear model analysis revealed no significant effect of compatibility condition (*b* = −0.02, 95% CI [−0.05, 0.01], *z* = −1.13, *p*_*os*_ = 0.74). The proportion of action repetitions did not significantly differ between the compatible (*M* = 0.50, *SD* = 0.07) and incompatible (*M* = 0.51, *SD* = 0.08) conditions. Therefore, Hypothesis 1 was not supported.

#### Recognition performance

For Hypothesis 2, we tested the effect of compatibility condition on recognition performance. Mean *d’* values by compatibility condition are depicted in Fig. 4B. Recognition performance was not significantly better in the compatible (*M* = 1.39, *SD* = 0.84) than in the incompatible (*M* = 1.37, *SD* = 0.82) condition (Δ*M* = 0.02, 95% CI [−0.06, 0.10], *t*(175) = 0.55, *p*_*os*_ = 0.29, *d* = 0.04, 95% CI [−0.11, 0.19]).^8^ Thus, Hypothesis 2 was not supported.

#### Recognition performance and action repetitions

For Hypothesis 3, we tested the effect of compatibility condition and action repetition on recognition performance. The generalized mixed linear model analysis revealed no significant main effect of compatibility condition (*b* = −0.01, 95% CI [−0.07, 0.05], *z* = −0.44, *p*_*ts*_ = 0.66). Recognition performance was not significantly better, and was actually significantly worse, given an action repetition than given an action alternation (*b* = −0.11, 95% CI [−0.17, −0.04], *z* = −3.32, *p*_*ts*_ < 0.001). There was further no significant interaction (*b* = 0.03, 95% CI [−0.03, 0.09], *z* = 1.00, *p*_*ts*_ = 0.32). Thus, Hypothesis 3 was not supported.

### Discussion

In Experiment 4, we kept the location of the effect identity constant across compatibility conditions and reduced the salience of the spatial movement of the box. As a result, participants no longer had a higher propensity to repeat an action from a previous prime phase in a later probe phase if they were re-presented with an effect identity that had been part of an action-effect compatible (vs. incompatible) episode in the prime phase. This suggests that the previously observed influence of AEC on action repetitions is attributable to E-E binding (i.e., participants retrieving the effect location and accordingly selecting their action in the probe phase), instead of to A-E binding (i.e., participants directly retrieving the key). Note again, however, that this conclusion rests on a null effect. However, we conducted a supplemental post-hoc analysis in which we jointly analyzed the data from Experiments 2 and 4. These experiments were very similar, differing only in the nature of the spatial effect feature. A generalized mixed linear model with experiment as an additional person-level covariate yielded a significant interaction between compatibility condition and experiment (*b* = 0.04, 95% CI [0.02, 0.06], *z* = 3.38, *p*_*ts*_ < 0.001). This suggests that the effect of compatibility condition did indeed differ between the experiments, lending further support to the claim that the influence of AEC on action repetitions is attributable to E-E binding (see Fig. 1). We again did not find evidence for an effect of AEC on long-term declarative memory for effect identity, nor an effect of action retrieval in the probe phase on long-term memory.

## General discussion

In four experiments, we investigated whether spatial action-effect compatibility (AEC) facilitates the retrieval of an action used to produce a certain effect (here: a box movement followed by a word) upon later reoccurrence of this effect identity. A second question was whether AEC generally facilitates long-term declarative memory for the effect identity. In a prime phase, participants could decide to press one of two keys, each assigned to a spatial movement of a box. The box then moved either spatially compatibly or incompatibly to the key press and a word appeared superimposed on the box at the end of its movement. In a later probe phase, effect words (i.e., effect identities) were re-presented, and participants had to spontaneously respond with a key press. Later, their memory for effect identities was probed in a free recall or recognition test.

In Experiments 1–3, we consistently found a higher propensity of participants to repeat an action from the prime phase in the later probe phase if they were re-presented with an effect identity that had been presented at a location compatible with their previous action in the prime phase than if they were re-presented with an effect identity that had been presented at a location incompatible with their action in the prime phase. These findings could be explained by AEC (i.e., the compatibility between the action location and the effect movement) facilitating action-effect (A-E) binding (i.e., the binding between action and effect identity) in long-term memory and therefore also action retrieval upon repetition of the effect identity. However, they can also be explained by the effect identity becoming associated with spatial effect features, that is, by effect-effect (E-E) binding (i.e., the binding between effect location and effect identity). Upon reoccurrence of the effect identity, these spatial effect features could have been retrieved, and participants chose an action in the probe phase that matched these spatial effect features.

Our findings from Experiments 3 and 4 do indeed support this latter account. In Experiment 3, in which we introduced a neutral prime key that did not clearly (mis)match any spatial movement of the box, participants’ propensity to select an action matching the effect location in the probe phase occurred independent of the prime response type. This suggests that the effect of AEC can at least partly be accounted for by the retrieval of the effect location through E-E binding and does not (fully) reflect an effect of AEC proper. In addition, in Experiment 4, the effect of AEC disappeared when we reduced the influence of spatial effect features, lending further support for the assumption that it is mostly the retrieval of the effect location that drove the previously observed effect of AEC. Considering that Experiments 1–3 included a vertical effect movement, whereas Experiment 4 included effect rotation, one may ask whether the differences in results between the experiments may be attributed to spatial attention. For example, people tend to exhibit anticipatory saccades towards anticipated effect locations (Gouret & Pfeuffer, 2021; Pfeuffer et al., 2016). This may have benefitted effect encoding in compatible action-effect episodes in Experiments 1–3 compared to incongruent ones, whereas, given the fixed effect location in Experiment 4, such a benefit due to AEC may have diminished. In this case, however, one may argue that memory for the effect identities in incompatible action-effect episodes in Experiment 4 should be boosted compared to the previous experiments. This was not the case; descriptively recognition performance for effect identities in incompatible action-effect episodes was even slightly worse than in Experiments 2 and 3. It seems therefore unlikely that observed differences between experiments are (solely) driven by spatial attention.

These findings allow us to draw several conclusions about feature binding in long-term memory. First, they suggest that bindings between features of action-effect episodes (here primarily between different effect features) can persist in long-term memory. This extends previous research, which mostly investigated action-effect bindings for rather short time scales (Dutzi & Hommel, 2009; Hommel, 2004; Mocke et al., in press; Moeller et al., 2016; Moeller & Frings, 2022). It is also in line with studies demonstrating persisting binding for other feature types. Whitehead et al. (2022) found bindings between stimuli and control processes that were active during their encoding to persist for up to 5 min even after one-shot learning. However, such stimulus-control binding may differ from stimulus-action or action-effect binding observed in other research. Several studies observed persistent effects of stimulus-action binding given one-time coupling of stimulus and effect after several interleaving experimental trials (Dames et al., 2022) or (short) experimental blocks (Pfeuffer et al., 2017, 2018), though not after a temporal delay of one day (Pfeuffer et al., 2017). However, even with several interleaving experimental blocks, effects could still be attributed to short-term memory processes, such that, for example, the action-effect episode still being maintained in working memory, as the duration of each block was only about 4 s. Furthermore, Schreiner and Kunde (2024), who explicitly asked participants about these links, found evidence for action-effect binding after longer time periods of several minutes even after one-time coupling of action and effect, suggesting that action-effect bindings can persist as associations in declarative long-term memory (see also Horner & Henson, 2009). While these studies attempted to investigate feature bindings across longer time scales, our current study is unique given that it involved a more indirect test of action-effect relations after one-shot learning and after a relatively long time interval.

Importantly, theories of feature binding, such as the theory of event coding (Hommel et al., 2001) or the binding and retrieval in action control (BRAC) framework (Frings et al., 2020), assume several influencing factors on the binding and retrieval of features in episodes involving actions and perceptual effects (here, AEC would constitute a bottom-up influence on binding), but are not designed for long-term memory phenomena (cf. Frings et al., 2023). A newly proposed framework further suggested that, at least for actions that are performed in response to a stimulus, bindings between action and effect features are a precursor for the formation of associations in long-term memory (Frings et al., 2023). Our findings add to these theories by showing long-term memory contributions to feature binding even after single encounters of an action-effect episode. On a more practical note, our results suggest, at least in early learning, a dominance of binding the identity of an effect, such as the content of a window opening on a computer, to features of the action-effect episode that are more anchored in the environment, such as the location on the screen in which the window appears, rather than to the action (e.g., a mouse click) through which the effect identity was produced. This suggests that, if the target information to be conveyed is a link between such more external features, this information may well be conveyed through observational learning to a similar extent, without the necessity of the learner to perform an action. Such learning occurs quickly, and the relevant information can be conveyed with very limited training. If the target information to be conveyed, however, is a link between the action and the effect, learning appears to be slower and may require a higher number of repetitions of action-effect episodes to yield reliable learning outcomes (cf. Schreiner & Kunde, 2025b; see also Frings et al., 2023).

Second, the current data are mostly in line with bindings between effect features and less with bindings between action and effect features (see Fig. 1). E-E binding serves as a mechanistic explanation for the obtained effect of AEC in Experiments 1–3. Due to the constant word locations, this E-E binding is not possible in Experiment 4, which explains the lack of an AEC effect. This is somehow at odds with the results by Mocke et al. (in press) who found an influence of AEC on action repetition in a sequential prime probe paradigm using box rotations, as in our Experiment 4. Note, however, that in this study, the retrieval of actions by effect identities occurred within a very short time frame after the prime episode, whereas in the present experiments, this delay was much longer. Together, this implies that bindings among effect features might be longer lasting or retrievable for longer than bindings among action and effect features, at least after one-shot learning and when probed indirectly. Possibly, that is because two features of the same environmental event tend to have a higher temporal contiguity than a feature of an action and a feature of the subsequent environmental effect, especially with longer action-effect delays. This is generally in line with findings by Schreiner and Kunde (2024), which suggest a stronger influence of E-E than A-E binding in long-term memory for one-shot learning, while also finding evidence for long-term influences of A-E binding (which may grow stronger with associative learning, cf. Elsner & Hommel, 2001; Hommel et al., 2003). In fact, one may wonder whether an action is needed at all to produce the binding of effect identity and effect location. Note, however, that Schreiner and Kunde (2024) used object images as effect stimuli, which may facilitate action-effect binding compared to words, as used in the current study (cf. Frings et al., 2023). In addition, sufficient strength of A-E bindings may be necessary to detect possible modulations by AEC. On a practical note, our results suggest that the influence of AEC on action retention, while generally possible also for longer time intervals (Schreiner & Kunde, 2024), may be quite limited to short time periods (see Mocke et al., in press). AEC may therefore be primarily important for situations in which action-effect episodes repeat in close temporal succession, such as when driving a car, scrolling on a webpage, or operating a machine such as an assembly belt. Especially in cases in which accurate responses to certain environmental signals are important, such as driving, these results suggest that systems should be designed to maximize AEC. For other situations that emphasize the long-term retention of action-effect links, AEC may not be of such primary importance and be subordinate to other factors. However, it is important to note that, beyond memory considerations, AEC exerts a strong influence on the sense of agency (Ebert & Wegner, 2010; Liesner et al., 2020; Schreiner et al., 2024a), which also plays an important role in human–machine interactions (Wen & Imamizu, 2022; Yu et al., 2024), for example, by affecting cognitive engagement.

Third, in Schreiner and Kunde (2024) participants were explicitly instructed to reproduce the action they used to produce an effect earlier after indicating that they recognized an effect identity. In the current research, however, participants were asked to respond spontaneously, without specific instructions about what to reproduce, and irrespective of whether they recognized the re-presented effect identity or not. Therefore, the contribution of implicit (nondeclarative) memory processes to actions in the probe phase may be stronger than in the study by Schreiner and Kunde (2024), which likely involved mostly explicit (declarative) memory processes. Together, the results suggest that the relative weight of A-E and E-E bindings in long-term memory does not change substantially when additionally considering the contribution of implicit memory processes, or it may even slightly shift further towards E-E binding. This relates to an ongoing discussion regarding the contribution of explicit and implicit memory processes to feature binding in action-effect episodes (e.g., Custers, 2023; Greenwald, 1970; Janczyk et al., 2024; Kunde & Janczyk, 2024; Sun et al., 2020). Indeed, results by Schreiner and Kunde (2025b) suggest the (early) learning of action-effect relations to be primarily supported by explicit memory. This is in line with our findings.

Regarding declarative long-term memory for effect identities, we found evidence for a facilitating effect of AEC in Experiment 1, using a free recall test, but only for effect identities that were presented in both the prime and probe phase, suggesting that they need to be sufficiently well encoded for the effect to emerge (or here, to avoid floor effects). This finding extends previous findings, in which an effect of AEC on long-term memory for effect identities using a recognition test was found (Hon & Teo, 2025; Hon & Yeo, 2021; Schreiner et al., 2024a). However, we did not observe this effect in Experiments 2–4, in which we too used a recognition test. This is inconsistent with these previous findings (but consistent with Tsuji & Imaizumi, 2022). However, the blocked prime-probe paradigm we adopted in the current study differed considerably from these previous studies and may not be optimal for investigating the effect of AEC on long-term memory for effect identity. Most notably, the current research included an intermittent probe phase in which participants were re-presented with effect identities between the prime and recognition phase. Possible explanations for the absence of an effect of AEC on recognition in the current research may be: (i) An increased temporal spacing between the prime and recognition phase reducing the effect size, possibly suggesting the effect does not stay stable over longer time periods (but note that we did observe it in free recall). (ii) The repeated presentation of effect identity (occurring in both the prime and probe phase) eliminating differences in memory performance due to AEC. This may disproportionately benefit memory for effect identities in incompatible action-effect episodes. In the free recall test of Experiment 1, performance was generally much lower than recognition performance in the other experiments, which may explain why we observed an effect in Experiment 1. (iii) The intermittent probe phase causing interference, eliminating the effect of AEC on recognition performance. The prime and probe phase differed in several effect features (e.g., a white vs. gray color of the box or the word located at the top or bottom vs. center of the screen). Given the re-presentation of the effect identity in the probe phase this may have activated several incompatible retrieval cues, which may have hindered recognition. In the free recall test, however, the effect identity was not re-presented, and memory retrieval may have thus relied less on such, possibly competing, retrieval cues. Given that we did not find an effect of AEC on recognition performance and the relatively low free recall performance in Experiment 1, it is perhaps not surprising that we also did not observe an effect of action repetitions in the probe phase on long-term memory.

## Data Availability

All raw data, analyses scripts, and experimental material are openly available at the Open Science Framework (Schreiner et al., 2024b; https://osf.io/b6h4s/). For all experiments we report how we determined our sample size, all data exclusions, all manipulations, and all measures in the studies, and the studies follow JARS (Appelbaum et al., 2018). All experiments’ designs, hypotheses, and analysis plans were preregistered (Experiment 1: 10.17605/OSF.IO/ZAW62; Experiment 2: 10.17605/OSF.IO/J5Y4K; Experiment 3: 10.17605/OSF.IO/ZCGPK; Experiment 4: 10.17605/OSF.IO/32FEJ).

## Appendix

### Word stimuli

Arch, axle, bank, barn, bead, beak, bean, beef, belt, bone, boot, bowl, brow, bulb, cape, case, cave, cell, chin, clam, coke, comb, cord, cork, corn, crab, crop, crow, deck, desk, dice, dish, dock, dome, dune, file, fish, foam, foil, foot, fork, gate, gear, glue, goat, golf, heel, herd, hill, hood, hoof, horn, inch, iron, jeep, jury, knot, lamp, lane, limb, lime, line, list, lock, maid, mall, mask, mast, meat, mill, monk, moss, nail, navy, nose, oven, pear, peel, pier, pine, pipe, pole, pond, pope, pork, port, post, rail, reel, rice, road, robe, rock, roof, root, sail, salt, seat, sole, soya, stem, tail, tape, taxi, tent, text, tool, tram, tube, turf, twig, tyre, veil, vein, vest, wing, wire, wood, yeti, yolk.

## Footnotes

1 In fact, in many cases, a defining feature of AEC is a shared dimension of action and effect features. For example, when moving a digital object on the computer one may press the up arrow key, prompting an upward movement of the object. Thus, action and effect location overlap (“up” for both the action and the effect location).

2 Nevertheless, we additionally tested the hypotheses based on aggregated data using paired-samples *t*-tests or repeated-measures analyses of variance if such analyses could in principle be appropriately applied. The results of these aggregated analyses generally converged with those of the mixed linear model analyses we report in all experiments. The only exception was that, when testing Hypothesis 1 in Experiment 3 with a paired-samples *t*-test, the effect of compatibility condition was not significant (*p*_*os*_ =.052). However, this may be attributed to a reduced statistical power of this aggregated analysis.

3 While excluding random slopes may pose problems (e.g., Barr et al., 2013), models with additional random slopes yielded almost identical model fit as the models with only random intercepts, with the latter generally exhibiting a slightly better fit. In addition, the qualitative interpretation of the results was not affected by the in- or exclusion of random slopes. This was the case for all experiments. Considering that the inclusion of random slopes that are not supported by the data can also pose problems (e.g., Oberauer, 2022), such as a loss in statistical power, we report the results of the models with only random intercepts.

4 Indeed, when we computed the individual differences in proportions of selecting the upper (or left, Experiment 4) response key between the prime and probe phase, the standard deviation of these differences varied between 0.15 and 0.19 across experiments, suggesting some degree of interindividual variability.

5 One participant reported an invalid age. The age of this participant was treated as a missing value.

6 One may argue that, also in the neutral condition, the effect direction may still be perceived as incompatible with the participant’s keypress. However, we argue that in the neutral condition, participants likely do not have an expectation regarding a certain effect direction, which qualitatively differs from the incompatible condition, in which they do have an expectation that is then violated. Therefore, the neutral condition may represent non-compatibility rather than incompatibility.

7 While it may be tempting to supplementarily compute Bayes factors after non-significant frequentist test results, this approach is problematic, as the resulting Bayes factors tend to be biased towards evidence for the null hypothesis (Schreiner & Kunde, 2025a; see also Dienes, 2024).

8 Note that there were considerable deviations from normality. However, a non-parametric paired-samples Wilcoxon signed-rank test yielded the same result (*V* = 5040.5, *p*_*os*_ =.30).

## Code availability

All analysis and experiment code are openly available at the Open Science Framework (Schreiner et al., 2024b; https://osf.io/b6h4s/). Data were analyzed using R (version 4.3.1, R Core Team, 2023). Specific packages used for the analyses are reported in the data analysis sections. Further packages that were used are the tidyverse collection (version 2.0.0, Wickham et al., 2019), here (version 1.0.1, Müller, 2020), papaja (version 0.1.2, Aust & Barth, 2023), egg (version 0.4.5, Auguie, 2019), and schoRsch (version 1.10, Pfister & Janczyk, 2022).
